# Tuberculous Abscess of the Chest Wall Simulate Pyogenic Abscess

**DOI:** 10.1155/2015/195412

**Published:** 2015-11-05

**Authors:** Lantam Sonhaye, Abdoulatif Amadou, Faré Gnandi-Piou, Kouméabalo Assih, Mazamaesso Tchaou, Bérésa Kolou, Kokou Adambounou, Bidamin N'Timon, Lama Agoda-Koussema, Komlavi Adjenou, Koffi N'Dakena

**Affiliations:** ^1^Radiology Department, CHU, 18BP216 Lomé, Togo; ^2^Surgery Department, CHU, Kara, Togo; ^3^Radiology Department, CHU, Kara, Togo

## Abstract

The chest wall tuberculosis abscesses is rare. We present a case of a 27-year-old immunocompetent male who presented chest wall abscesses. Imaging (chest radiographic, ultrasound, and computed tomography) and Ziehl-Neelsen staining demonstrated chest wall tuberculosis abscesses.

## 1. Introduction

Chest wall tuberculosis (TB) is rare localization of extrapulmonary TB and accounts for 1–5% of all musculoskeletal TB, which itself is very rare [1].

Three mechanisms are described in the pathogenesis of chest wall abscess: direct extension from pleural or pulmonary parenchymal disease, hematogenous dissemination of a dormant tuberculous focus, or direct extension from lymphadenitis of the chest wall [2].

Primary tuberculosis of the chest wall is rare and diagnosis in most of the cases is demanding and effortful because the lesions grossly simulate pyogenic abscess or tumour.

Here we are presenting a case of tuberculous abscess of the chest wall simulate pyogenic abscess of a 27-year-old immunocompetent.

## 2. Case Report

A 27-year-old male presented with painful swelling over left chest region over 5th to 8th rib area. The swelling had gradually increased in size.

He had no history of fever, weight loss, cough, haemoptysis, expectoration, or any past history of tuberculosis. Family history was noncontributory.

**Figure 1:**
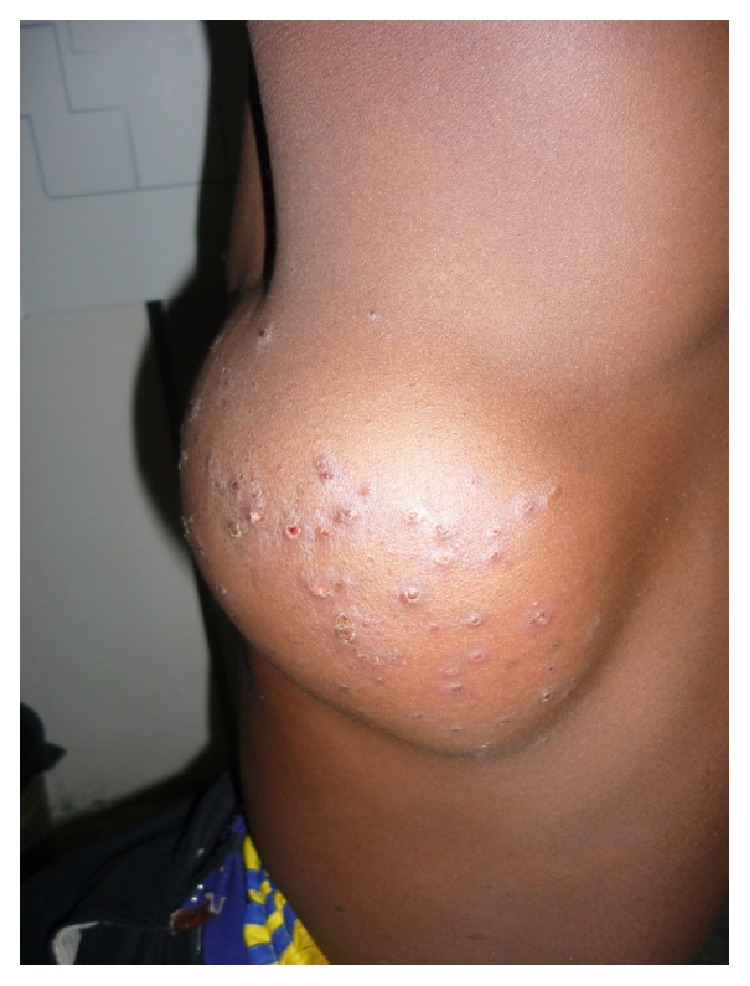
Showing large solitary well defined swelling with pustules on the skin on left chest wall over 5th to 8th ribs area.

On examination, the patient was average built, afebrile, and with normal pulse and blood pressure. Respiratory system examination was normal. Local examination revealed a large swelling of 17 × 13 cm over the left chest region over 5th to 8th rib area, with ill-defined borders. The lesion was soft, fluctuating, tender, warm, movable, and not attached to underlying structures; the swelling presented the pustules on the skin (Figure 1).

His haemogram, liver, and renal functions were within reference ranges. Serology for HIV was nonreactive.

**Figure 2:**
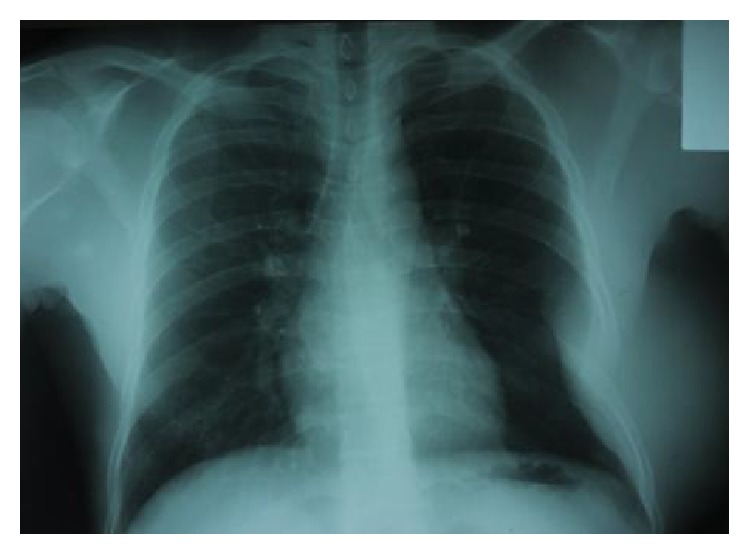
Chest X-ray showing unilateral peripheral opacity.

Chest X-ray showed unilateral peripheral opacity (Figure 2).

**Figure 3:**
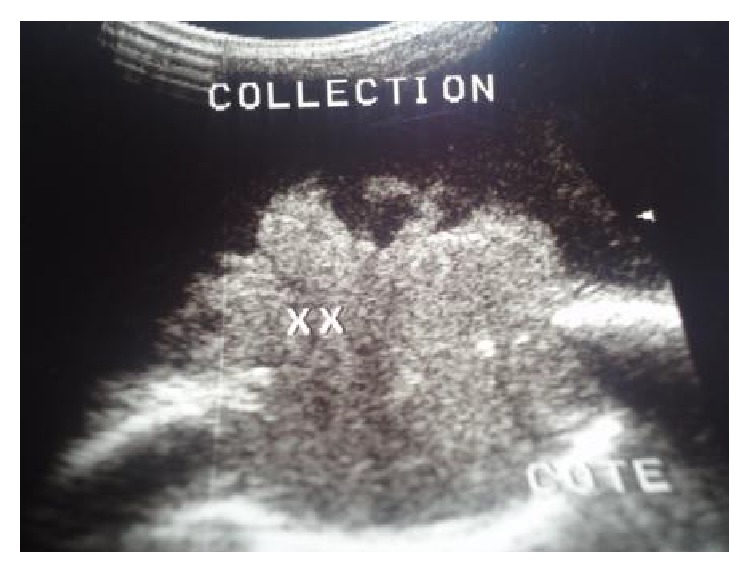
Ultrasonography showing hypoechoic lesion with dense internal echoes and destruction of rib.

**Figure 4:**
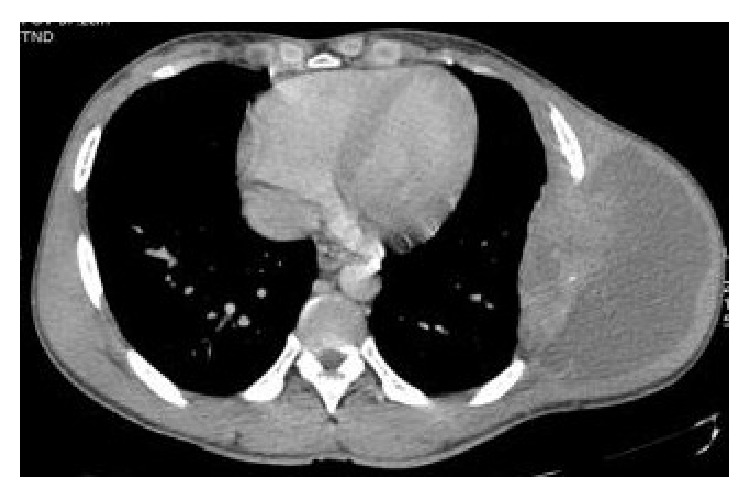
Axial Computed Tomography image at lesion level in mediastinal window showing well loculated hypodense collection in the left chest wall with peripheral enhancement.

**Figure 5:**
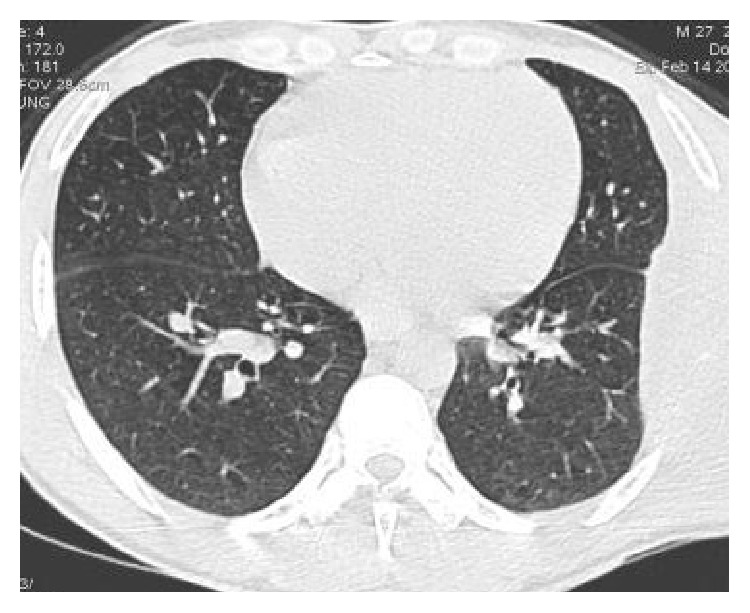
Axial Computed Tomography image at lesion level in lung window showing no evidence of lung parenchymal lesion.

**Figure 6:**
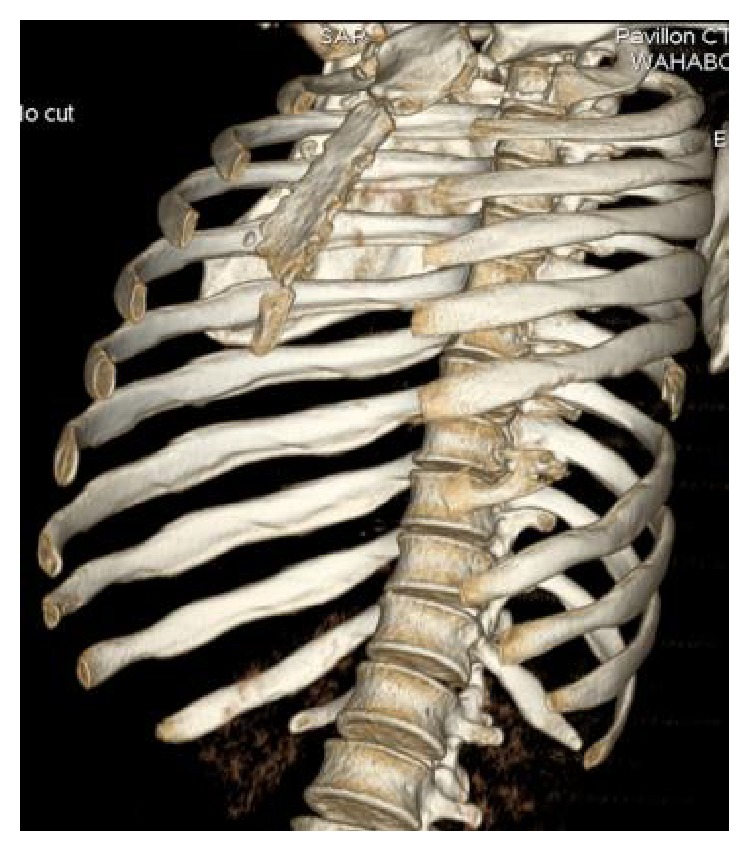
Computed tomography 3D reformation showing destruction of the 7th rib.

Ultrasonography showed well defined hypoechoic lesion with dense internal echoes (Figure 3). Both plain and contrast enhanced computed tomography revealed loculated hypodense collection of 17 × 13 cm in the left chest wall with peripheral enhancement (Figure 4) and destruction of the 7th rib (Figure 5). There was no evidence of either lung parenchymal lesion (Figure 6) or mediastinal lymphadenopathy; the abdomen was within normal limits.

Urine sample was negative for Acid Fast Bacilli (AFB).

Fine needle aspiration from swelling was done and yielded pus. Aerobic bacterial culture of the pus was sterile and cytology was negative for malignancy. But pus on Ziehl-Neelsen staining demonstrated AFB.

Surgical procedures were drainage and wide debridement with rib resection. The patient was put on antituberculous medical treatment for 6 months.

On follow-up after 12 months, patient had responded well to treatment and the abscess resolved completely.

## 3. Discussion

Primary tuberculosis of the chest wall is rare and diagnosis in most of the cases is demanding and effortful because the lesions grossly simulate pyogenic abscess or tumour.

In this particular case, the swelling appeared to be a pyogenic abscess, as a result of direct inoculation because the skin of the swelling had pustules on the skin due to the local treatment administration of herbalist in low socioeconomic country.

Tuberculous abscesses of the chest wall can involve the sternum, costochondral junctions, ribs shafts, costovertebral joints, and the vertebrae. They usually occur as a solitary lesion, most frequently at the margins of the sternum and in the shafts of the ribs [3].

The preoperative diagnosis of primary tuberculosis of the chest wall is difficult [2]. An initial needle aspiration guided by ultrasonography of the swelling is necessary to first establish a diagnosis and second to exclude other diagnoses such as malignancy and other infectious diseases [4]. But needle aspiration alone is often not reliable, and surgical biopsy is usually required to establish a definite diagnosis [5].

Radiologic imaging is important in the assessment of chest wall tuberculosis abscess, particularly for determining anatomic origin and extent, response to therapy, and recurrence [6]. Computed tomography (CT) imaging plays an important role in the evaluation of this chest wall abnormality due to its excellent spatial resolution, including depiction of both osseous and soft-tissue structures. Multidetector Computed Tomography (MDCT) enables imaging of a large tissue volume in a short acquisition time, reducing the effect of respiratory motion in the thorax. On the other hand, CT may reveal mineralization and bony involvement with a higher sensibility and specificity when compared to MRI or ultrasound with a significant impact in the differential of these conditions [7].

Treatment of chest wall tuberculosis is controversial and there is no consensus on the optimal treatment. Some authors suggest that medical treatment alone is effective; others believe that aggressive debridement with primary closure in addition to medical therapy is required to prevent recurrence or formation of a draining sinus [8]. Cho et al. [2] recommended preoperative and postoperative tuberculosis medication and complete resection of chest wall mass including any suspicious rib.

Although WHO recommends a standard 6-month regiment, according to clinical presentation, bacillary load, and response to antituberculous medical therapy, the treatment can be extended up to 9–12 months [9].
